# Nutrients Uptake and Accumulation in Plant Parts of Fragrant *Rosa* Species Irrigated with Treated and Untreated Wastewater

**DOI:** 10.3390/plants11091260

**Published:** 2022-05-06

**Authors:** Muhammad Ahsan, Muhammad Nafees, Muhammad Amin, Fahim Nawaz, Aasma Tufail, Hasan Sardar, Shadi Shokralla, Eman A. Mahmoud, Ahmed M. El-Sabrout, Hosam O. Elansary

**Affiliations:** 1 Department of Horticultural Sciences, The Islamia University of Bahawalpur, Bahawalpur 63100, Pakistan; muhammad.nafees@iub.edu.pk (M.N.); m.amin@iub.edu.pk (M.A.); 2 Department of Agronomy, MNS, University of Agriculture, Multan 66000, Pakistan; fahim5382@gmail.com; 3 Department of Botany, Division of Science and Technology, University of Education, Lahore 54000, Pakistan; aasmatufail@gmail.com; 4 Department of Horticultural Sciences, Bahauddin Zakaria University, Multan 60800, Pakistan; hasan.sardar@bzu.edu.pk; 5 Centre for Biodiversity Genomics, University of Guelph, Guelph, ON N1G 2W1, Canada; sshokral@uoguelph.ca; 6 Department of Food Industries, Faculty of Agriculture, Damietta University, Damietta 34511, Egypt; emanmail2005@yahoo.com; 7 Department of Applied Entomology and Zoology, Faculty of Agriculture (EL-Shatby), Alexandria University, Alexandria 21545, Egypt; elsabroutahmed@alexu.edu.eg; 8 Plant Production Department, College of Food & Agriculture Sciences, King Saud University, Riyadh 11451, Saudi Arabia

**Keywords:** environmental pollution, macronutrients, ornamental crop, water purification

## Abstract

Water scarcity has critically augmented the need for the exploration of alternative irrigation sources mainly in water-scarce regions. This water scarcity has put tremendous pressure on the agri-based economy of countries such as Pakistan. The reuse of sewage wastewater has been appearing as the only alternative water source, which can lessen our dependence upon freshwater (FW). The current study aimed to scrutinize the influence of treated wastewater (TWW) and untreated wastewater (UTWW) irrigation on the nutrient (N, P, K, Ca, and Na) concentration in different plant parts, i.e., roots, stems, leaves, and flowers, of four scented *Rosa* species (*R. bourboniana, R. centifolia, R.* Gruss-an-telpitz, and *R. damascena*) during the first week of 2018 to the last week of 2019. The experiment was arranged according to the two-factor factorial arrangement i.e., factor I was the irrigation source, while factor II was the *Rosa* species. The experimental water analysis showed that mineral and chemical concentrations in FW and TWW were within permissible limits of national environmental quality standards (NEQSs) for wastewater. The UTWW of this study possessed a higher electrical conductivity (EC), chemical oxygen demand (COD), biological oxygen demand (BOD), total nitrogen (TN), and metals (Cd, Co, and Pb) than recommended levels. The results revealed that P, K, Ca, and Na contents significantly increased in all studied plant parts of *Rosa* species as the duration of irrigation with TWW and UTWW increased and vice versa in the case of N contents, while the ratio of N content elevation by applying TWW and UTWW were also not increased compared to other studied nutrients. The nutrients (except Ca) were found as maximum in all plant parts with UTWW compared to FW and TWW irrigation in roses. These stimulations were accredited to the presence of higher essential nutrients and some metals in UTWW. This experiment confirmed the disparities in nutrient contents of scented *Rosa* species due to the different absorbability of each element in every plant part. Regarding the nutrient accumulation in rose plant tissues, the results of the present study confirm that untreated wastewater must be treated to some extent to grow scented roses where water is scarce.

## 1. Introduction

Climate change is one of the most solemn challenges that pose a threat to the livelihoods of poor people belonging to climate-sensitive and agriculture-reliant developing nations of the world [1]. Pakistan is one of those countries where the lethal impacts of water shortage, due to climate change, are not limited to the production of food crops but have extensive consequences for ornamental crop productions. Water, being the most vital natural resource for life, has been a key issue on the global agenda for many years [2]. The world’s freshwater resources are dwindling very rapidly due to extensive irrigation demands for agricultural lands. Thus, irrigation water resources must be utilized with superior efficacy, and the use of nonconventional water sources such as treated wastewater (TWW) and untreated wastewater (UTWW) should be encouraged [3]. Consequently, wastewater reuse is a major strength for agricultural drives and the most vital choice to manage water scarcities [4]. 

For centuries, TWW and UTWW have been utilized for irrigation purposes in Vietnam, China, and Mexico [5], but in Pakistan, Israel, Jordan, Saudi Arabia, and India, the utilization of marginal-quality wastewater has been common practice for the last decade due to the dearth of freshwater [6]. The application of wastewater is a trustworthy irrigation source for agricultural crop production in peri-urban areas where high-value perishable crops can be cultivated near the consumer markets [7]. As wastewater is a rich source of macronutrients, i.e., nitrogen (N), phosphorus (P), and potassium (K), the extra charges for chemical fertilizers can be reduced significantly [8]. Such marginal-quality wastewater can be applied as an irrigation source in less fertile soils where it may deliver nutrients required for plant growth and development and its supply is even and steadfast [9]. It can enhance the Na, Fe, and Ca concentration in the soil and can elevate the organic matter concentration of soil up to 60% [10]. Marinho et al. [11] observed a significant buildup of N, P, and K in plant parts of rosebushes by the application of reclaimed wastewater. Modanloo and Darvishi [12] stated that urban wastewater is an asset to supply macronutrients in horticultural crops, which significantly reduced the cost of artificial fertilizer application. Morgan et al. [13] also showed a higher macronutrient level in leaves of citrus plants due to the application of reclaimed wastewater, which enhanced the fruit quality and other horticultural characteristics. This efficient wastewater utilization can create self-employment, produce more food, and generate revenue [14]. According to Tymchuk et al. [15], there has been a propensity to decrease the utilization of potassium, nitrogenous, and phosphate fertilizers in many countries of the world, which is linked with a reduction in the natural resources required to produce these fertilizers. This issue can be resolved by the usage of sewage wastewater containing N, P, and K [15]. Gryshko and Korinovskaya [16] stated that wastewater, on average, may contain N up to 1–3%, P up to 1–5%, and K up to 0.2–0.7%, so it is an admirable resource of these macronutrients. 

Constant municipal and industrial wastewater irrigation may cause the accretion of heavy metals in soils as well as in plants [17,18]. These toxic metals have achieved significant consideration in the world because of key environmental pollutants by its toxic nature and accrual capacity [19]. Some heavy metals, at the miniscule level, can disturb metabolic activities and are considered as critical for life [2]. However, if their concentration crosses the standard limit in living organisms, these metals may provoke many physiological and biochemical malfunctioning [20]. These heavy metals accruals in plants and soils by wastewater result in the adulteration of soil and are disparaging to animals and humans as their stockpile in the environment disturbs food safety [21]. 

Marginal-quality wastewater consumption for the production of medicinal as well as ornamental plants (i.e., roses) can shield the tarnishing food web [22]. Oil-containing ornamental and medicinal plants contain no adulteration, i.e., heavy metals in oils [23,24]. As ornamental plants are not utilized concurrently for food purposes, the severe treatment of sewage wastewater is not recommended [25]. Being regarded as the “queen of flowers”, rose has a lion share as an ornamental crop in the global floricultural market [6]. It is a perennial flowering woody plant with more than 20,000 cultivars cultivated worldwide and is used as cut flowers, interior decoration, garden plants, and, occasionally, for food purposes [26]. In Pakistan, only four scented and oil-bearing species of roses are cultivated, i.e., *Rosa bourboniana*, *R.* Gruss-an-teplitz, *R. damascena,* and *R. centifolia*. Freshwater is mostly utilized for irrigation purposes to these roses and, hence, very rare information is accessible to our farmers about the influence of marginal-quality TWW and UTWW. It was first detailed in a comparative study to check the performance of four fragrant rose species under these marginal-quality wastewaters irrigation in Pakistan. Therefore, the current experiment aimed to enumerate the build-up of N, P, K, Na, and Ca concentrations in roots, stems, leaves, and flowers of four scented *Rosa* species that were cultivated using TWW and UTWW for irrigation in a peri-urban area of Faisalabad, Pakistan. 

## 2. Results

### Nutrient Concentration in Plant Parts

**Figure 1:**
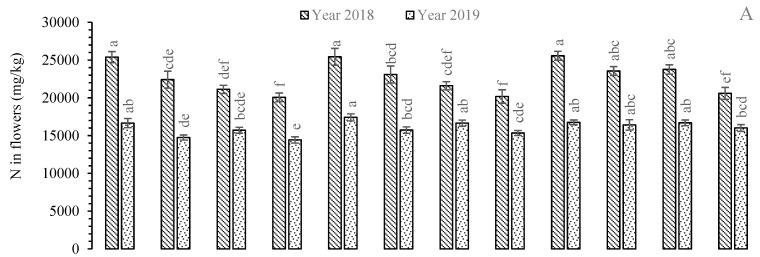

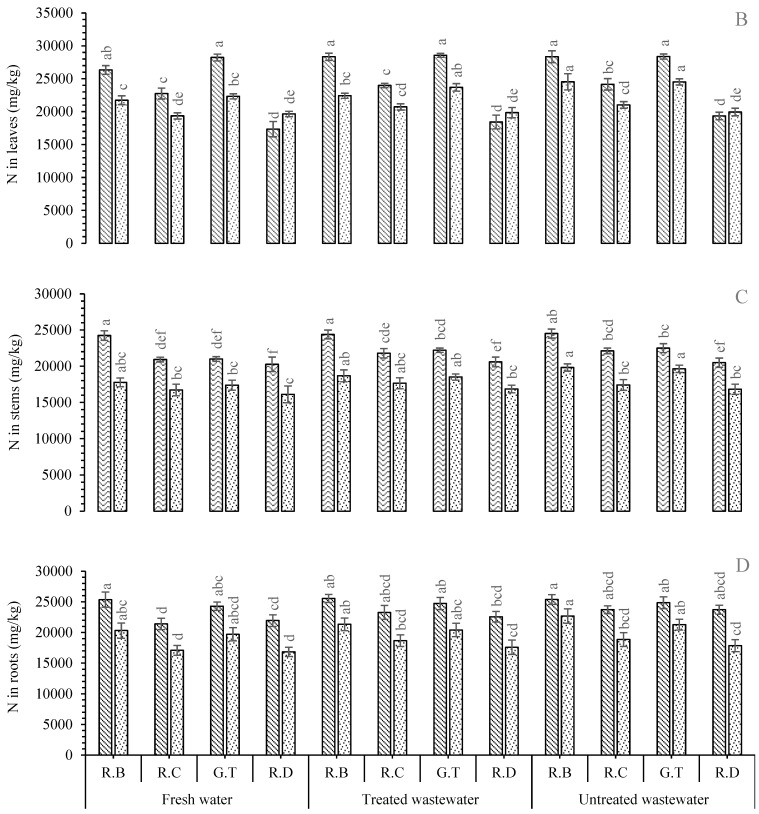
Mean concentration of N in plant parts, i.e., flowers (**A**), leaves (**B**), stems (**C**), and roots (**D**) of *Rosa* species under different irrigations. Each vertical bar represents the average of three replicates. Different letters indicate significant differences among treatments at *p* ≤ 0.05 according to least significant difference test. R.B: *Rosa bourboniana*, R.C: *Rosa centifolia*, G.T: Gruss-an-teplitz, R.D: *Rosa damascena*.

All experimental rose species revealed a statistically significant (*p* < 0.05) effect on N concentration irrigated with TWW and UTWW during both years of study. The highest N level was recorded in the roots, stems, and flowers of *R. bourboniana,* while *R.* Gruss-an-teplitz contained the greatest N value in leaves during 2018 and 2019 (Figure 1). In the roots, the mean N concentration was increased 0.77% and 5.05% under TWW conditions and 0.1% and 11.68% under UTWW conditions compared to FW (control)-irrigated plants in 2018 and 2019, respectively. The smallest N concentrations were found in *R. centifolia* (2018) and in *R. damascena* (2019) under FW conditions. For stems, there were 0.59% and 5.1% enhancements under TWW and 1.14% and 11.55% increases in N concentration in *R. bourboniana* under UTWW during 2018 and 2019, respectively. The leaves of *R.* Gruss-an-teplitz showed 1.14% and 0.49% (during 2018), and 6.14% and 9.82% (during 2019) N increases in TWW and UTWW compared to FW-irrigated plants, respectively. In flowers, there were 0.17% and 0.69% increments during 2018, and 4.56% and 0.59% increases in N levels during 2019 in *R. bourboniana*. The smallest N concentrations in the flowers of *R.* Gruss-an-teplitz were exhibited under FW conditions, which were reduced by 2.27% and 12.51% during 2018, and 5.99% and 6.2% during 2019 under TWW and UTWW conditions, respectively. The overall trend of N concentration was found in leaves > flowers > roots > stems (Figure 1). 

**Figure 2:**
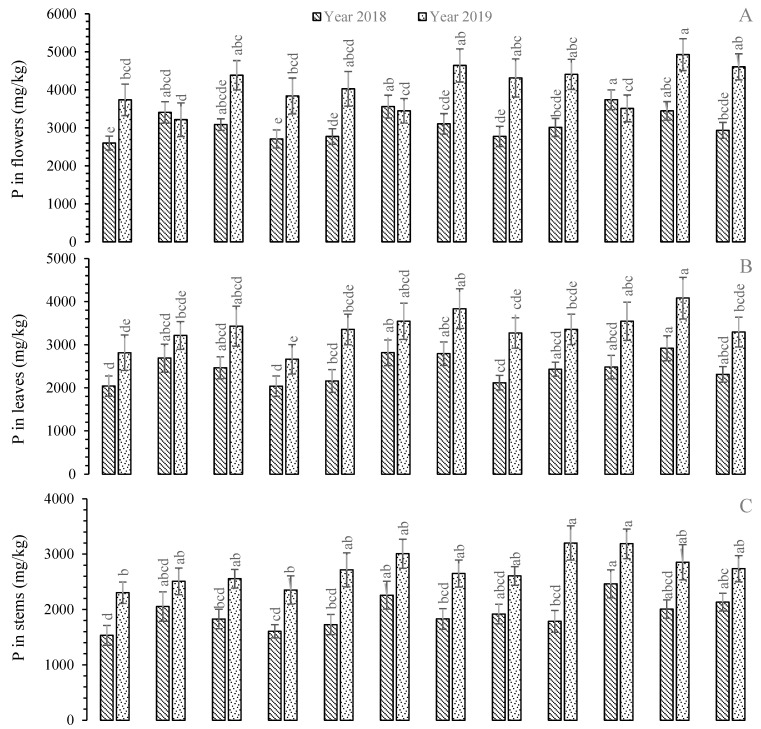

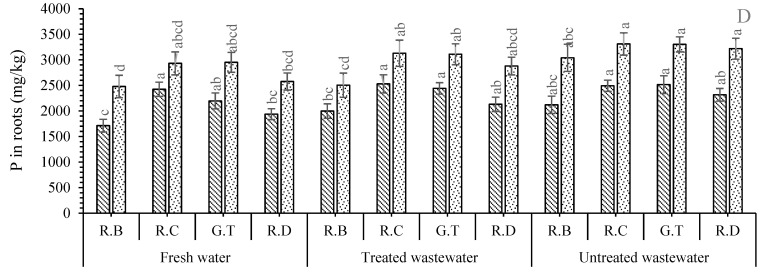
Mean concentration of P in plant parts, i.e., flowers (**A**), leaves (**B**), stems (**C**), and roots (**D**), of *Rosa* species under different irrigations. Each vertical bar represents the average of three replicates. Different letters indicate significant differences among treatments at *p* ≤ 0.05 according to least significant difference test. R.B: *Rosa bourboniana*, R.C: *Rosa centifolia*, G.T: Gruss-an-teplitz, R.D: *Rosa damascena*.

The greatest mean P concentration was found in the roots of *R. centifolia* under TWW and UTWW during 2018 and 2019, respectively, followed by *R.* Gruss-an-teplitz with UTWW (Figure 2). There were 4.34% and 12.96% increases in P levels with TWW (2018) and UTWW (2019) compared to FW (control), respectively. During 2018, the stems of *R. centifolia* contained the highest P level, while *R. bourboniana* contained the highest level during 2019 under UTWW. The P levels were enhanced by 19.99% and 39.06% under UTWW compared to FW-irrigated plants in *R. centifolia* and *R. bourboniana,* respectively. The lowest P concentrations were recorded in the roots and stems of *R. bourboniana* under FW during both years of the experiment. The leaves of *R.* Gruss-an-teplitz contained the greatest P concentration with UTWW, whereas the lowest value was found with *R. damascena* under FW during 2018 and 2019 (Figure 2). Increases of 18.38% (during 2018) and 18.99% (during 2019) were exhibited with UTWW compared to FW. The smallest P value with FW-irrigated plants was decreased up to −3.93% and −13.44% in 2018 and −22.88% and −23.63% in 2019 under TWW and UTWW, respectively. It was interesting to find that the P concentration was reduced (-7.77%) with *R. centifolia* under UTWW compared to FW-irrigated plant leaves during 2018. The flowers of *R. centifolia* and *R.* Gruss-an-teplitz contained the highest P level under UTWW during 2018 and 2019, respectively (Figure 2). The P concentrations were elevated by 9.74% (in 2018) and 12.37% (in 2019) in UTWW compared to FW-receiving plants. The smallest P level was recorded under FW with flowers of *R. bourboniana* (−6.69% TWW and −15.88% UTWW) and *R. centifolia* (−7.27% TWW and −9.21% UTWW). The overall trend of P level was found in flowers > leaves > roots > stems (Figure 2).

**Figure 3:**
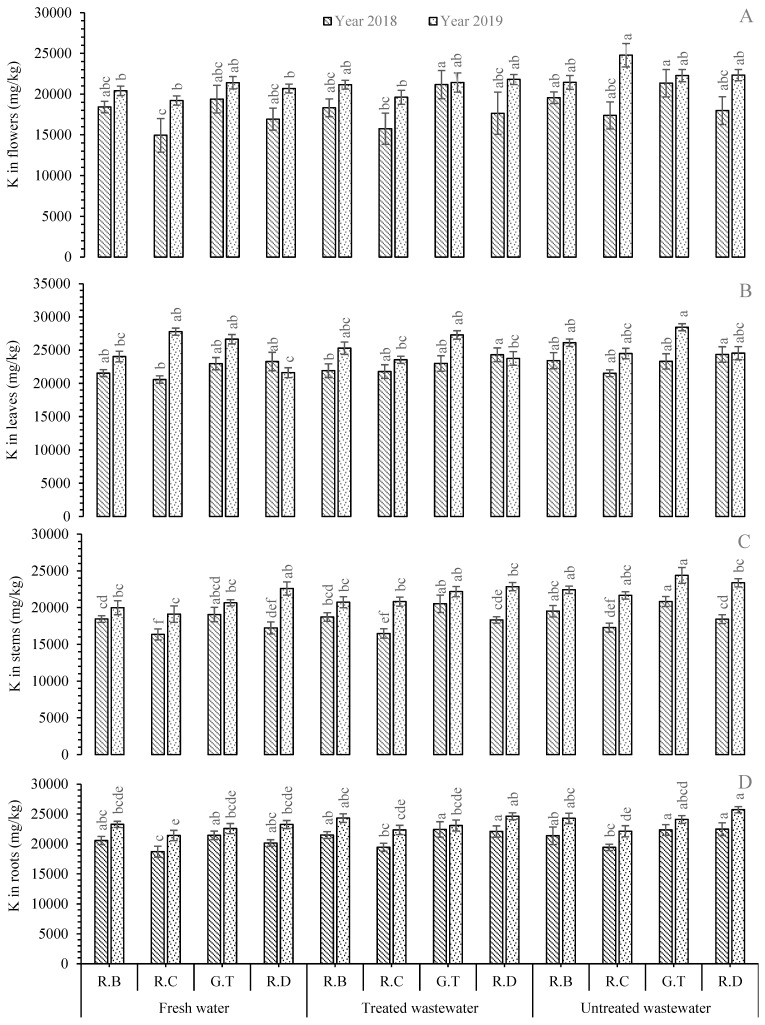
Mean concentration of K in plant parts, i.e., flowers (**A**), leaves (**B**), stems (**C**), and roots (**D**), of *Rosa* species under different irrigations. Each vertical bar represents the average of three replicates. Different letters indicate significant differences among treatments at *p* ≤ 0.05 according to least significant difference test. R.B: *Rosa bourboniana*, R.C: *Rosa centifolia*, G.T: Gruss-an-teplitz, R.D: *Rosa damascena*.

During 2018 and 2019, the greatest K concentration was recorded in the roots of *R. damascena* under UTWW, whereas the minimum K value was found in the roots of *R. centifolia* under FW (Figure 3). The K concentration exhibited increments of 11.52% and 10.59% under UTWW compared to FW-irrigated plants in 2018 and 2019, respectively. In the stems, the highest K concentration was observed with *R.* Gruss-an-teplitz under UTWW, while the lowest value was recorded under FW with the stems of *R. centifolia* during both experimental years. The K concentration exhibited decreases of 9.29% (during 2018) and 18.05% (during 2019) under FW compared to UTWW in *R.* Gruss-an-teplitz (Figure 3). During 2018, the greatest K concentration (+4.55%) was found in the leaves of *R. damascena* under UTWW followed by the same species under TWW (+4.38%). It was interesting to note that the leaves of *R.* Gruss-an-teplitz showed the maximum K level in 2019 under UTWW (+6.77%) followed by TWW (+2.41), while the minimum value was recorded in the leaves of *R. bourboniana* under FW irrigation (Figure 3). In flowers, the highest K level was recorded under UTWW (+10.11% than FW), followed by TWW (+9.18% than FW) in *R.* Gruss-an-teplitz during 2018. *R. centifolia* flowers contained the lowest K concentration under FW during the first experimental year. In 2019, the flowers of *R. centifolia* under UTWW possessed the highest K concentration followed by *R. damascena* under the same irrigation regime. It exhibited 28.93% and 7.97% increments compared to FW. The lowest K concentrations were found in the flowers of *R. centifolia* under FW irrigation, which were −2.07% and −28.93% lower than those of TWW and UTWW, respectively. The overall trend of K levels was found in leaves > roots > flower > stem (Figure 3).

**Figure 4:**
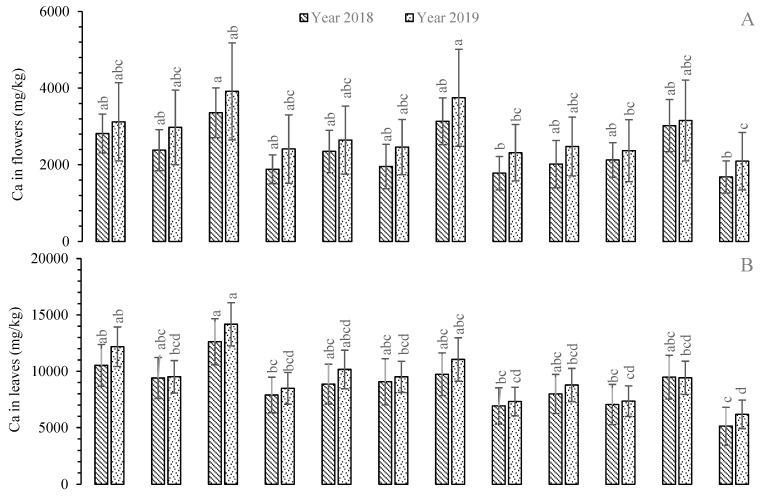

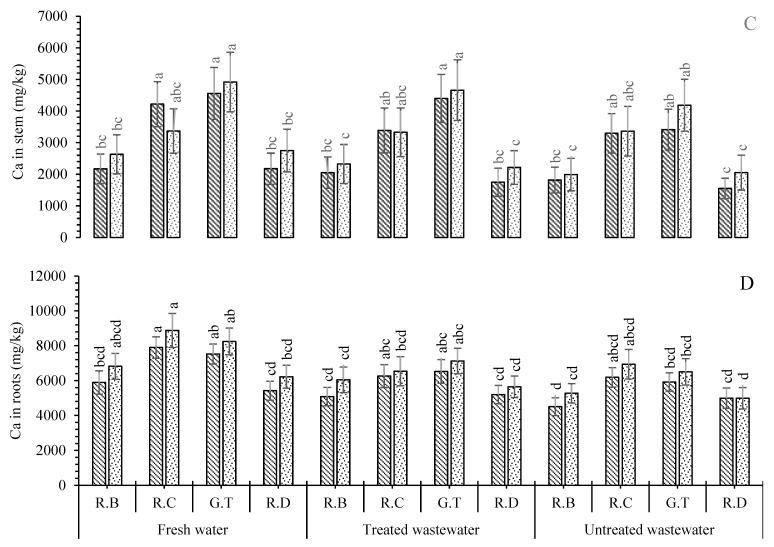
Mean concentration of Ca in plant parts, i.e., flowers (**A**), leaves (**B**), stems (**C**), and roots (**D**), of *Rosa* species under different irrigations. Each vertical bar represents the average of three replicates. Different letters indicate significant differences among treatments at *p* ≤ 0.05 according to least significant difference test. R.B: *Rosa bourboniana*, R.C: *Rosa centifolia*, G.T: Gruss-an-teplitz, R.D: *Rosa damascena*.

The mean Ca concentration in roots of *R. centifolia* under FW was greatest and statistically significant (*p* < 0.05) during 2018 and 2019. The Ca levels exhibited reductions of −20.71% and −21.65% in the roots of the same species irrigated with TWW and UTWW, respectively (Figure 4). The lowest Ca concentration was recorded in the roots of *R. bourboniana* and *R. damascena* under UTWW during both years of the experiment. These Ca values with UTWW were −13.72% and −23.55% lower than those of TWW and FW, respectively. In the stems, FW-irrigated plants of *R.* Gruss-an-teplitz contained the highest Ca level followed by the same species under TWW during 2018 and 2019. The Ca level exhibited reductions of −3.51 and −25.14% in 2018 and −5.22 and −14.91% in 2019 under TWW and UTWW, respectively, compared to FW. The minimum value was recorded in *R. damascena* during 2018 and in *R. bourboniana* during 2019 under UTWW-irrigated plant stems (Figure 4). The greatest Ca concentration was found in the leaves of *R.* Gruss-an-teplitz followed by *R. bourboniana* under FW irrigation during 2018 and 2019. In 2018, increases (with FW-irrigated plants) of +22.88% and +24.89% were exhibited in *R.* Gruss-an-teplitz, and increases of +15.78% and +24.06% were exhibited in *R. bourboniana* compared to TWW- and UTWW-irrigated plants, respectively. The lowest Ca concentrations were recorded in the leaves of *R. damascena* with UTWW during both years of the experiment. This Ca level decrease in *R. damascena* under UTWW was −34.98% during 2018 and −27.22% during 2019 compared to FW-irrigated plants of the same species (Figure 4). As with that in leaves, a similar trend was also observed in the flowers of *R.* Gruss-an-teplitz under FW followed by the same species under TWW and UTWW during 2018 and 2019. The Ca level exhibited increments of +6.47% and +9.99% during 2018 and +4.3% and +19.48% during 2019 under FW as compared to TWW and UTWW, respectively. The minimum Ca concentrations were observed in the flowers of *R. damascena* under UTWW, which were −10.71% and −13.21% lower than those of FW-irrigated plants during 2018 and 2019, respectively. The overall trend of Ca concentration was recorded in leaves > roots > stems > flowers (Figure 4).

**Figure 5:**
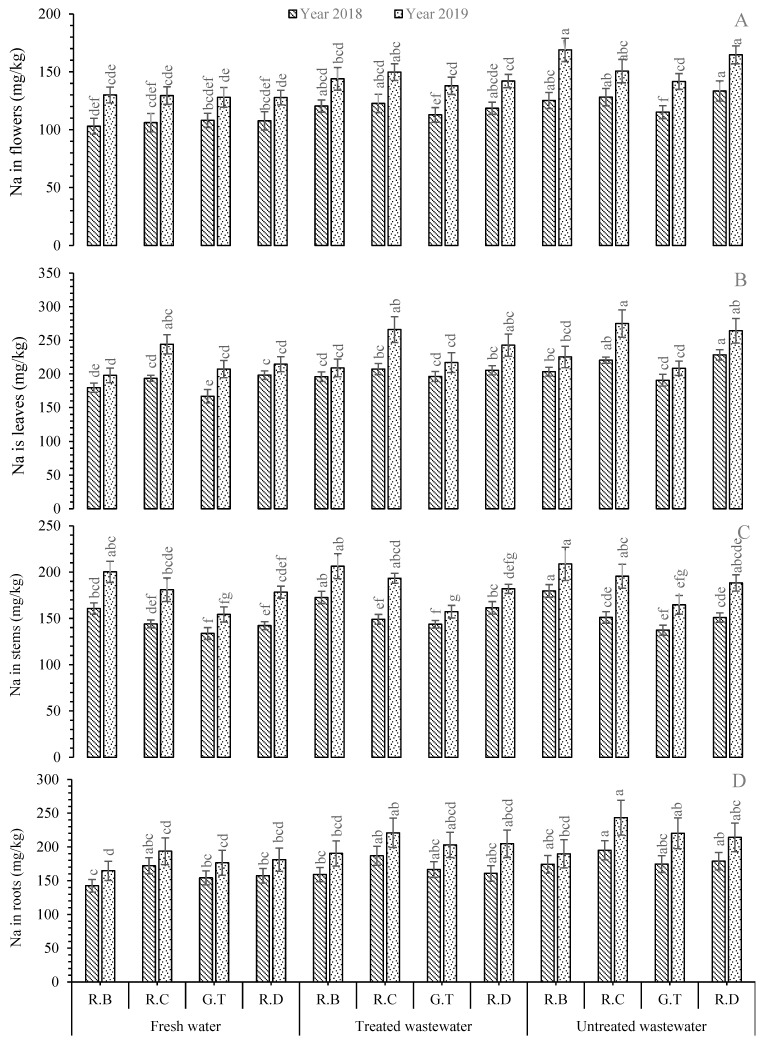
Mean concentration of Na in plant parts, i.e., flowers (**A**), leaves (**B**), stems (**C**), and roots (**D**), of *Rosa* species under different irrigations. Each vertical bar represents the average of three replicates. Different letters indicate significant differences among treatments at *p* ≤ 0.05 according to least significant difference test. R.B: *Rosa bourboniana*, R.C: *Rosa centifolia*, G.T: Gruss-an-teplitz, R.D: *Rosa damascena*.

The results showed that the greatest mean Na concentration was recorded in the roots of *R. centifolia* under UTWW followed by the same species under TWW during both years of the experiment. The Na content level exhibited elevations of +13.17% and +8.01% during 2018 and +24.83% and +15.1% during 2019 with UTWW and TWW, respectively, with *R. centifolia*. The lowest Na concentration was recorded with the roots of *R. bourboniana* under the FW irrigation source (Figure 5). The Na level in the stem was greatest with *R. bourboniana* UTWW during 2018 and 2019. This increment under UTWW was +11.84% and +4.25% compared to FW irrigation during 2018 and 2019, respectively (Figure 5). The lowest Na concentration was found with FW-irrigated plants of *R. damascena* (during 2018) and *R.* Gruss-an-teplitz (during 2019). In the case of the leaves of the *Rosa* species, the greatest mean Na level was found in *R. damascena* and *R. centifolia* under UTWW during 2018 and 2019, respectively. It had a +15.19% increase in *R. damascena* and +12.62% increase in *R. centifolia* compared to FW-irrigated plants. The lowest Na concentration was found in the leaves of *R. bourboniana* during 2018 and 2019 under FW (Figure 5). The greatest mean Na level in the flowers was found in *R. centifolia* during 2018, whereas in *R. bourboniana* during 2019, it was under UTWW-irrigated plants. This increase was +20.8% with *R. centifolia* and +29.92% with *R*. *bourboniana* under UTWW as compared to FW-irrigated plants. The lowest Na value was found under FW in the flowers of *R. bourboniana* and *R. damascena* during 2018 and 2019, respectively. The overall trend in Na concentration was noted in leaves > roots > stem > flowers (Figure 5).

## 3. Discussion

Roses have been categorized as salt-tolerant up to 3.8 dS/m [27], sensitive [1], and severely sensitive to salinity [28] with an EC level as low as 0.8–1.0 dS/m. Contrarily, Dyubeni et al. [29] also noted that some rose species could resist up to 6.0 dS/m without upsetting flower quantity and quality. This study showed a difference in response of *Rosa* species to various macronutrients and even their contents in different parts, i.e., root, stem, leaves, and flowers. Similar results were obtained by Alghobar and Suresha [30] where the concentrations of N, P, K, Ca, and Na were significantly elevated in different parts of tomato plants when irrigated with municipal wastewater as compared to freshwater-irrigated plant samples. Similar findings were also reported by Salehi and Tabari [31] when pine trees were irrigated with untreated urban wastewater for two years and found higher concentrations of EC, pH, Na, Mg, N, P, K, and Ca in roots and needle-shaped leaves of pine trees compared to well water-irrigated plants. These higher nutrient levels are due to higher salts and other macronutrients levels in wastewaters that were used continuously for irrigation to these plants [32]. 

Nitrogen is an essential macronutrient that plays an important role in plant biochemistry, contributing to the formation of compounds vital to plant life such as proteins, amino acids, and nucleic acid [33]. Nitrogen is used particularly in nitrate form by plants, which is a soluble compound. Smirnoff and Stewart [34] stated that plants of the Rosaceae family are experts in the utilization of N in nitrate form. Wastewater is a cheap source of N in nitrate form for the crop plants [35]. It was observed that the N contents in plant parts varied due to the different irrigation regimes during this study, while a significant elevation of N was noted in the leaves and flowers than roots of *Rosa* species irrigated with both wastewater treatments. This significant buildup was the consequence of N supply by TWW and UTWW as its absorption by plant parts, especially leaves [6]. This upsurge can be accredited to N migration from the roots to leaves and flowers [36]. The application of wastewater for a long-term duration led to a reduction in crop nutrient status due to nutrient leaching and reduction in crop size [37]. Similar findings were also obtained in this study where the N contents were higher in all plant parts for the first year than the second year of experiment. When utilizing TWW and UTWW for irrigation, the increase in crop productivity is linked with the type of plants and concentration of nutrients in wastewaters [2]. Plants of *R. bourboniana* contained a higher N level than other species, while *R. centifolia* possessed the lowest. This was due to species variation, which affects crop yield traits including the plant morphology, flower quality, yield, and composition of elements in plant parts [38].

Phosphorus is the second-most essential crop nutrient after N [39]. It plays a key role in plant functioning and is a vital component of proteins, sugar phosphate, phospholipids, enzymes, and energy-rich phosphate compounds [36]. According to Yuan et al. [40], municipal wastewater contains 1–5% of P, which is a cheap source compared to other artificial fertilizers. Contrary to N, the concentration of P was highest in *R. centifolia* than other *Rosa* species during this experiment. Both wastewaters (TWW and UTWW) caused elevations in P contents in the plant parts of roses under study. These results are in line with the findings of Al-Karaki et al. [41] who stated that TWW and UTWW are large sources of P contents at the plant nutrient level. Shahbazzadeh and Amirineiad [42] compared the P concentration in horticultural crops cultivated under municipal wastewater and well water and observed a significantly higher quantity of P in wastewater-irrigated plants than well-watered horticultural plants. Furthermore, the long-term application of wastewater adds more P levels to plants than short-term wastewater application [38]. Similar findings were also recorded during this study where the P contents were higher in all parts of *Rosa* species during 2019 as compared to 2018. As the P contents were within permissible levels with all irrigation regimes, UTWW contained the highest P level compared to FW and TWW. Chiou [43] stated that TWW leads to a higher accumulation of P in soil and plants, which made it hazardous for the growth of food crops, so this treated wastewater should be applied to ornamental crops. 

Potassium availability may result due to wastewater irrigation, which may correspond to or be in excess to plant necessities [44]. Potassium is involved in many physio-biochemical functions such as the regulation of transportation of stomata in plants [45] and enhances plant tolerance to different stresses such as drought and salinity [8]. Similarly, K is involved in the activation of some enzymes responsible for plant metabolism [46]. Studies have shown that the K concentration increases in plant parts as the K level rises from FW to TWW and UTWW. This increment was credited with the steady supply and obtainability of these nutrients to plants after every irrigation with TWW and UTWW [6,47]. As with our study, Nicolas et al. [48] observed higher contents of macronutrients (N, P, and K) in plant parts due to the continuous irrigation of wastewater as compared to ground water. This nutrient uptake elevation in *Rosa* species might be due to a sufficient amount of these nutrients in the plant root zone through wastewater irrigation [49]. It could also be due to the high transformation rate of soil nutrients via microbiological activities in the soil, which led to an elevated bioavailability of nutrients to plant species and consequently resulted in higher contents in plant biomass [50]. There was no significant change in K contents with *Rosa centifolia* compared to other species, due to the change in irrigation regime in this study. These findings are in contrast with the results of Dysko et al. [51] who reported P contents in tomato plants when salts and nutrient contents were increased in wastewaters. This might be due to nutrient variations from plant species to species, the concentration of heavy metals in water sources, exposure time, the environment/medium in which plants were grown, and different parts of each plant depending on the nutrient absorption rate from the soil by the rose plants [52]. 

In addition to three major macronutrients N, P, and K in TWW and UTWW as elucidated earlier, the presence of other essential nutrients such as Ca (essential component of cell wall) might have also played a key role in cell divisions, regulating ions transportation [53], and its presence in wastewater could also have proved valuable for plant growth and development [54]. Previous findings have shown that TWW and UTWW (to some extent) improves the biomass of microbes, soil water holding capacity (WHC), organic matter contents in soil (OM), and porosity, which leads to favoring plant growth and increases biomass [55,56,57,58]. In the same line, Wang et al. [59] noted that after the application of TWW, OM and WHC increase in the soil, which helps plants to attain nutrients. Thus, the use of wastewaters for irrigation increases the fertility status of soil [48,60] and can offer soil with nutrients (N, P, K, Ca, Mg, Na, etc.). In the present study, Ca contents showed a decreasing trend due to the application of TWW and UTWW, which contained higher metal concentrations in all plant parts of *Rosa* species. Similar results were also found by Al-Absi et al. [61] who showed that wastewater due to the presence of heavy metals caused Ca deficiency in some crops. This decreasing value to Ca under TWW and UTWW irrigation might be due to higher Mg and other nutrients (especially K) contents, which could have acted antagonistically to the Ca uptake by the roots [60]. According to documentation of McDonnell et al. [62], an elevation in K level in plants is accompanied by a reduction in the contents Ca and Na. 

Sodium is a vital beneficial element, which may kindle growth by expansion of the cell in addition to plant water balancing [8]. Wastewaters used in this experiment contained Na levels within permissible limits according to national environmental quality standards (NEQSs) of wastewater for irrigation to plants. In the present research, Na contents increased for the second year as compared to the first year of study. This could be due to the higher concentration of Na in both wastewater treatments compared to FW, and the continued application of this wastewater elevated the Na buildup in all parts of *Rosa* species. Bedbabis et al. [63] illustrated that the Na level was found to be elevated under TWW than FW. Batarseh [64] found higher contents of Na and K accompanied by higher ECs in TWW and UTWW. These results were contrary with the findings of McDonnell et al. [62], who stated that an excess of K in plant parts leads to a reduction in Na concentration. Alghobar and Suresha [65] stated that UTWW and TWW significantly increased the concentration of Na in plant tissues of rice crops. He verified our results and further explained that Na contents increased with the increase in wastewater with the order of UTWW > TWW > GW in plant parts. This could be related to the quantity of sufficient nutrient elements present in the wastewater that were accumulated in plant parts. 

## 4. Materials and Methods

### 4.1. Research Area Description, Plant Collection, and Experimental Layout

The current research study was carried out from the first week of January 2018 to the last week of December 2019 at the Research Area of Institute of Horticultural Sciences, University of Agriculture, Faisalabad, Pakistan. The research region was in a semi-arid climate with fright annual rainfall. The mean monthly minimum and maximum temperature range (°C) are illustrated in Figure S1. The experimental field vicinity was irrigated by sewage wastewater from the University. At depths of 15 cm and 30 cm, 16 sites were randomly selected for soil sampling. These composited soil samples were analyzed by standard procedures proposed by the U.S. Salinity Laboratory Staff [66], and data are expressed in Table 1.

Irrigation waters and scented *Rosa* species were two treatment factors of this study. Vigorous and healthy cuttings of two-years-old rose species were collected from the Department of Floricultural Research, Faisalabad. These 20 cm long cuttings were planted in pits (0.3048 m^2^ depth) with 1.22 m plant-to-plant distances according to the randomized complete block design (RCBD) with two-factor factorial arrangements with three replicates. These cuttings were irrigated with freshwater (FW), treated wastewater (TWW), and untreated wastewater (UTWW). Irrigation was applied after every 06–08 days during November to March, whereas this interval was reduced to 03–05 days from April to October depending on weather conditions. 

### 4.2. Decontamination of Wastewater and Analysis

Contaminated raw wastewater was treated by the process of solar purification to recover its physio-chemical qualities [69,70]. Raw sewage wastewater was treated in three large plastic tanks with 1550 gallons of water storage capability in three steps. The first tank was filled with raw wastewater via a pipe whose opening was covered with a sieve of 6 mm holes as a preliminary treatment to stop the grits and exclude source solid materials often found in untreated wastewater [71]. For sunlight penetration, the upper portion of the tank was kept uncovered as a natural purification process. Wastewater was stored for five days in this tank for the elimination of settleable organic and inorganic material by sedimentation and removal of floating material by skimming. This primary treated wastewater was shifted to the second tank (positioned 2.5′ away and 2′ below the first tank) for sedimentation of tiny particles and further purification for five days. Water treated in the second tank was shifted to the third tank for further sedimentation for the next five days. Water obtained after 15 days of the purification process was applied to plants as treated wastewater. According to standard methods proposed by Eaton et al. [72], the physico-chemistries of experimental waters and various nutrients and chemicals were quantified using inductively coupled plasma optical emission spectroscopy (ICP-OES, Optima 2100-DV Perkin Elmer, USA) at the Nuclear Institute of Agriculture and Biology (NIAB), Faisalabad.

Water samples (FW, TWW, and UTWW) were analyzed at ICP-OES. There were higher EC, BOD, and COD levels in samples of both wastewaters, while the nitrogen (N) and metals (Cd, Co, Pb) concentrations were above the standard limits set by national environmental quality standards (NEQSs) under UTWW. The samples of FW possessed values within recommended levels (Table 2). 

### 4.3. Determination of Nutrient Elements

#### Estimation of Nutrients in Plant Parts of Selected Rosa Species

About 100 g samples of roots, stems, leaves, and flowers of *Rosa* species were collected and dried in the shade and an electrical oven for 72 h at 70 °C. These dried samples were taken and grinded to a fine powder for determination of N, P, K, Ca, and Na levels in plant parts. Total N, P, and K concentrations were determined according to the method suggested by Chapman and Parker [73]. Potassium, Ca, and Na contents were determined by using the flame photometric analysis (410 Sherwood flame photometer, Cambridge, UK) suggested by Anonymous [74].

### 4.4. Statistical Analysis

By using the all-experimental data, an analysis of variance (ANOVA) was performed by using computer program STATISTIX (8.1). Treatment factors (irrigation water and *Rosa* species) means were compared according to the least significant difference (LSD) test at the 5% level of probability [75]. 

## 5. Conclusions

Treated wastewater and untreated wastewater significantly increased the nutrient concentration (except Ca contents, which were higher with FW irrigation) in the studied plant parts of all fragrant roses. Untreated wastewater possessed and transferred the highest nutrient (N, K, Ca, and Na) concentrations in the leaves, while the P level was maximum in flowers compared to other plant parts of *Rosa* species. Based on these findings, variations in nutrient absorbance and transportation status in different parts of fragrant roses were demonstrated. Furthermore, the application of TWW and UTWW for fragrant roses can be suggested, but the unceasing monitoring and management of toxic substances in underground water is a precondition to save freshwater sources and ultimately the environment. Long-term research also needs to be performed, even for these sweet-scented *Rosa* species, as the present experiment was based only on the results from 2 years.

## Figures and Tables

**Table 1:** Composition and analysis of soil before experiment.

Sr. No.	Characteristics	Unit	Value		IASS *
1	Texture		Clay loam soil		
			00–15cm	15–30 cm	
2	pH		8.2	8.2	4–8.5
3	EC	dS m^−1^	2.54	2.49	4
4	Organic Matter	%	1.12	1.18	>0.86
5	Nitrogen	mg/kg	0.041	0.041	---
6	Phosphorus	mg/kg	10.5	9.5	>7.0
7	Potassium	mg/kg	194	134	>80
8	Lead	mg/kg	3.16	3.32	6.0
9	Cadmium	mg/kg	0.04	0.05	1.0
10	Nickel	mg/kg	0.36	0.34	20
11	Zinc	mg/kg	5.28	3.6	10
12	Copper	mg/kg	3.04	2.3	6.0

Source: * International Agricultural Soil Standards [67,68].

**Table 2:** Analysis of freshwater, treated wastewater, and untreated wastewater.

Sr. No.	Parameters	Untreated Wastewater	Treated Wastewater	Freshwater	NEQS
1	EC (dS/m)	2.13	1.43	0.13	1.5
2	pH	8.31	7.58	7.22	6–9.2
3	Color	Greyish	Rust Brown	-	-
4	Turbidity	155	29.12	43	-
5	Hardness (mg/dm^3^)	536	416	184	-
6	DO (mg/dm^3^)	1.36	2.38	4	-
7	BOD (mg/dm^3^)	432	267	-	260
8	COD (mg/dm^3^)	558	481	-	450
9	TDS (mg/dm^3^)	1678	1281	218	3000
10	SS (mg/dm^3^)	1.1	0.15	0.8	-
11	Total Solids (mg/dm^3^)	1372	982	218	-
12	TSS (mg/dm^3^)	194	63	17	400
13	Chlorides (mg/dm^3^)	436	290	138	1000
14	Cadmium (mg/dm^3^)	0.013	0.01	0.001	0.01
15	Nickel (mg/dm^3^)	0.12	0.08	0.10	1.0
16	Arsenic (mg/dm^3^)	0.005	0.004	ND	1.0
17	Zinc (mg/dm^3^)	3.48	2.62	0.18	5.0
18	Potassium (mg/dm^3^)	34.6	21.71	7.61	-
19	Lead (mg/dm^3^)	0.66	0.42	0.021	0.5
20	Iron (mg/dm^3^)	4.82	3.47	0.32	5.0
21	Cobalt (mg/dm^3^)	0.079	0.029	0.17	0.05
22	Copper (mg/dm^3^)	0.24	0.13	0.05	0.5
23	Chromium (mg/dm^3^)	0.98	0.73	0.04	1.0
24	Calcium (mg/dm^3^)	57.35	39.72	28.1	200
26	Sodium (mg/dm^3^)	236.91	178.23	36.47	250
27	Magnesium (mg/dm^3^)	63	47	30	150
28	Phosphorus (mg/dm^3^)	2.49	1.76	0.39	15
29	Total Nitrogen (mg/dm^3^)	6.10	4.87	3.84	5.0

NEQS: National Environmental Quality Standards for municipal wastewater of Pakistan; Standard value of NEQS; EC: Electrical conductivity; DO: Dissolved Oxygen; BOD: Biological Oxygen Demand; COD: Chemical Oxygen Demand; TDS: Total Dissolved Solids; SS: Settleable Solids; TSS: Total Suspended Solids: ND: Not Detected. NEQS source: [68].

## Data Availability

All data are available within this publication.

## Supplementary Materials

The following are available online at https://www.mdpi.com/article/10.3390/plants11091260/s1, Figure S1: Mean monthly minimum and maximum temperature (°C) during the experimental period.

## References

[1] Ahsan M., Younis A., Nafees M., Tufail A., Shakeel Q., Raheel M., Nawaz F., Jaskani M.J., Amin M., Sajid M. (2021). Marginal quality water arbitrated essential oil concentration in metal hoarded flower petals of scented roses. Ecotoxicol. Environ. Saf..

[2] Hashem M.S., Qi X. (2021). Treated Wastewater Irrigation—A Review. Water.

[3] Rizzo L., Gernjak W., Krzeminski P., Malato S., McArdell C., Perez J., Schaar H., Fatta-Kassinos D. (2020). Best available technologies and treatment trains to address current challenges in urban wastewater reuse for irrigation of crops in EU countries. Sci. Total Environ..

[4] Ghernaout D., Elboughdiri N., Al Arni S. (2019). Water Reuse (WR): Dares, restrictions, and trends. Appl. Eng..

[5] Shuval H.I., Adin A., Fattal B., Rawitz E., Yekutiel P. (1986). Wastewater Irrigation in Developing Countries: Health Effects and Technical Solutions.

[6] Ahsan M., Younis A., Jaskani M.J., Tufail A., Riaz A., Schwinghamer T., Tariq U., Nawaz F. (2018). Heavy metal accumulation imparts structural differences in fragrant *Rosa* species irrigated with marginal quality water. Ecotoxicol. Environ. Saf..

[7] Ahsan M., Younis A., Jaskani M.J., Tariq U., Shaheen M.R., Tufail A., Sherani J., Nawaz F. (2019). Anatomical changes in stem of scented Rosa spp. in response to heavy metal accumulation under wastewater treatment. Int. J. Agric. Biol..

[8] Iqbal S., Inam A., Inam A., Ashfaque F., Sahay S. (2017). Potassium and waste water interaction in the regulation of photosynthetic capacity, ascorbic acid and capsaicin in chilli (*Capsicum annuum* L.) plant. Agric. Water Manag..

[9] Chen X.W., Tsz-Fung W.J., Mo W.Y., Man Y.B., Wang-Wai Ng C., Wong M.H. (2015). Ecological performance of the restored south east new territories (sent) landfill in Hong Kong (2000–2012). Land Degrad. Dev..

[10] U.S. Agency for International Development (2004). Guidelines for Water Reuse.

[11] Marinho L.E.O., Tonetti A.L., Stefanutti R., Filho B.C. (2013). Application of reclaimed wastewater in the irrigation of rosebushes. Water Air Soil Pollut..

[12] Modanloo M., Darvishi H.H. (2015). Nitrogen application and irrigation with purified urban wastewater effect on NPK accumulation in Fenugreek (*Trigonella foenum* L.). Walia J..

[13] Morgan K.T., Wheaton T.A., Parsons L.R., Castle W.S. (2008). Effects of reclaimed municipal wastewater on horticultural characteristics, fruit quality, and soil and leaf mineral concentration of citrus. HortScience.

[14] Khaleel R.I., Ismail N., Ibrahim M.H. (2013). The impact of wastewater treatments on seed germination and biochemical parameter of *Abelmoschus esculentus* L.. Soc. Behav. Sci..

[15] Tymchuk I., Shkvirko O., Sakalova H., Malovany M., Dabizhuk T., Shevchuk O., Matviichuk O., Vasylinych T. (2020). Wastewater a Source of Nutrients for Crops Growth and Development. J. Ecol. Eng..

[16] Gryshko V.M., Korinovskaya O.N. (2015). Influence of organo-mineral fertilizers on the basis of precipitation of sewage on micromycetes cenosis. Gruntoznavstvo.

[17] Aftab N., Saleem K., Khan A.H.A., Butt T.A., Mirza C.R., Hussain J., Farooq G., Tahir A., Yousaf S., Zafar M.I. (2021). *Cosmos sulphureus* Cav. is more tolerant to lead than copper and chromium in hydroponics system. Int. J. Environ. Sci. Technol..

[18] Ali S., Zohaib A., Muhammad R., Ihsan E.Z., Ilkay Y., Aydın Ü., Mohamed M., Abdel D., May B.J., Mirza H. (2020). Application of floating aquatic plants in phytoremediation of heavy metals polluted water: A review. Sustainability.

[19] Karahan F., Ozyigit I.I., Saracoglu I.A., Yalcin I.E., Ozyigit A.H., Ilcim A. (2020). Heavy metal levels and mineral nutrient status in different parts of various medicinal plants collected from Eastern Mediterranean Region of Turkey. Biol. Trace Elem. Res..

[20] Leblebici Z., Kar M. (2020). Heavy metals accumulation in vegetables irrigated with different water sources and their human daily intake in Nevsehir. J. Agric. Sci. Technol..

[21] Sarwar T., Muhammad S., Natasha, Khalid S., Shah A.H., Ahmad N., Naeem M.A., ul Haq Z., Murtaza B., Bakhat H.F. (2020). Quantification and risk assessment of heavy metal build-up in soil–plant system after irrigation with untreated city wastewater in Vehari, Pakistan. Environ. Geochem. Health.

[22] Khan A.H.A., Amna K., Cyrus R.M., Tayyab A.B., Rocío B., Basit A., Mazhar I., Sohail Y. (2021). Ornamental plants for the phytoremediation of heavy metals: Present knowledge and future perspectives. Environ. Res..

[23] Bernstein N., Chaimovitch D., Dudai N. (2009). Effect of irrigation with secondary treated effluent on essential oil, antioxidant activity, and phenolic compounds in oregano and rosemary. Agron. J..

[24] Darvishi H.H., Manshouri M., Sedghi H., Jahromi S.H.M. (2010). Irrigation influence by treated domestic wastewater instead of agronomical water on essential oil yield of basil (*Ocimum basilicum* L.). Afr. J. Microbiol. Res..

[25] Elsokkary I.H., Aboukila A.F. (2020). Beneficial additive values of wastewater irrigation of two aromatic plants grown in low fertile soil. Water Sci..

[26] Mahmood S., Reza M.R., Hossain M.G., Hauser B. (2018). Response of cytokinins on in vitro shoot multiplication of Rose cv. Frisco. J. Agric. Sci. Technol..

[27] Kotuby-Amacher J., Koeing R., Kitchen B. (2000). Salinity and Plant Tolerance. Utah State Uni. Ext.. http://www.extension.usu.edu/publica/agpubs/agso03.pdf.

[28] Western Australia Department of Agriculture (2003). Salinity Tolerance Chart. http://www.staneyo.com/news_files/water/salinity_chart.html.

[29] Dyubeni L., Mayekiso B., Magwa M.L. (2012). A comparative study on essential oil yield and composition of rose-scented geranium (P.c.v. Rose) commercially grown on three different sites of the Amathole region in the Eastern Cape, South Africa. Afr. J. Agric. Res..

[30] Alghobar M.A., Suresha S. (2015). Evaluation of metal accumulation in soil and tomatoes irrigated with sewage water from Mysore city, Karnataka, India. J. Saudi Soc. Agri. Sci..

[31] Salehi A., Tabari M. (2014). Nutritional properties of soil and leaves of Tehran pine trees irrigated with urban sewage. Environ. Sci. Technol..

[32] De Carlo L., Battilani A., Solimando D., Caputo M.C. (2020). Application of time-lapse ERT to determine the impact of using brackish wastewater for maize irrigation. J. Hydrol..

[33] Disciglio G., Gatta G., Libutti A., Gagliardi A., Carlucci A., Lops F., Cibelli F., Tarantino A. (2015). Effects of irrigation with treated agro-industrial wastewater on soil chemical characteristics and fungal populations during processing tomato crop cycle. J. Soil Sci. Plant Nutr..

[34] Smirnoff N., Stewart G.R. (1985). Nitrate assimilation and translocation by higher plants: Comparative physiology and ecological consequences. Physiol. Plant..

[35] Edelstein M., Ben-Hur M. (2018). Heavy metals and metalloids: Sources, risks and strategies to reduce their accumulation in horticultural crops. Sci. Hortic..

[36] Soufan W., Okla M.K., Al-Ghamdi A.A. (2019). Effects of irrigation with treated wastewater or well water on the nutrient concentration of two alfalfa (*Medicago sativa* L.) cultivars in Riyadh, Saudi Arabia. Agronomy.

[37] Mok H.-F., Barker S.F., Hamilton A.J. (2014). A probabilistic quantitative microbial risk assessment model of norovirus disease burden from wastewater irrigation of vegetables in Shepparton, Australia. Water Res..

[38] Malhotra H., Vandana S., Sharma S., Pandey R., Hasanuzzaman M., Fujita M., Oku H., Nahar K., Hawrylak-Nowak B. (2018). Phosphorus nutrition: Plant growth in response to deficiency and excess. Plant Nutrients and Abiotic Stress Tolerance.

[39] Rajasekar M., Udhaya-Nandhini D., Swaminathan V., Balakrishnan K. (2017). A review on role of macro nutrients on production and quality of vegetables. Int. J. Chem. Stud..

[40] Yuan H., Lu T., Wang Y., Chen Y., Lei T. (2016). Sewage sludge biochar: Nutrient composition and its effect on the leaching of soil nutrients. Geoderma.

[41] Al-Karaki G.N. (2011). Utilization of treated sewage wastewater for green forage production in a hydroponic system. Emir. J. Food Agric..

[42] Shahbazzadeh R., Amirinejad A.A. (2018). Effects of raw municipal wastewater on soil physical quality and biological yield of wheat (case study: Harsin). Iran. J. Soil Water Res..

[43] Chiou R.J. (2008). Risk assessment and loading capacity of reclaimed wastewater to be reused for agricultural irrigation. Environ. Monit. Assess..

[44] Arienzo M., Christen E.W., Quayle W., Kumar A. (2009). A review of the fate of potassium in the soil-plant system after land application of wastewaters. J. Hazard. Mat..

[45] Al-Suhaibani N., Seleiman M.F., El-Hendawy S., Abdella K., Alotaibi M., Alderfasi A. (2021). Integrative effects of treated wastewater and synthetic fertilizers on productivity, energy characteristics, and elements uptake of potential energy crops in an arid agro-ecosystem. Agronomy.

[46] Tak H.I., Babalola O.O., Huyser M.H., Inam A. (2013). Urban wastewater irrigation and its effect on growth: Photosynthesis and yield of chickpea under different doses of potassium. Soil Sci. Plant Nutr..

[47] Mkhinini M., Boughattas I., Alphonse V., Livet A., Gıustı-Mıller S., Bannı M., Bousserrhıne N. (2020). Heavy metal accumulation and changes in soil enzymes activities and bacterial functional diversity under long-term treated wastewater irrigation in East Central region of Tunisia (Monastir governorate). Agric. Water Manag..

[48] Nicolas E., Alarcón J.J., Mounzer O., Pedrero F., Nortes P.A., Alcobendas R., Romero-Trigueros C., Bayona J.M., MaestreValero J.F. (2016). Long-term physiological and agronomic responses of mandarin trees to irrigation with saline reclaimed water. Agric. Water Manag..

[49] Chojnacka K., Witek-Krowiak A., Moustakas K., Skrzypczak D., Mikula K., Loizidou M. (2020). A transition from conventional irrigation to fertigation with reclaimed wastewater: Prospects and challenges. Renew. Sustain. Energy Rev..

[50] Mansir I., Oertlé E., Choukr-Allah R. (2021). Evaluation of the Performance and Quality of Wastewater Treated by M’zar Plant in Agadir, Morocco. Water.

[51] Dysko J., Kaniszewski S., Kowalczyk W. (2015). Lignite as a new medium in soilless cultivation of tomato. J. Elem..

[52] Jilani M.I., Ahmad M.I., Hanif R., Nadeem R., Hanif M.A., Khan M.A., Ahmad I., Iqbal T. (2012). Proximate analysis and mineral profile of three elite cultivars of *Rosa hybrida* flowers. Pak. J. Bot..

[53] Manohara B., Belagali S.L. (2014). Characterization of essential nutrients and heavy metals during municipal solid waste composting. Int. J. Innov. Res. Sci. Eng. Technol..

[54] Akhtar N., Inam A., Inam A., Khan N.A. (2012). Effects of city wastewater on the characteristics of wheat with varying doses of nitrogen, phosphorus, and potassium. Recent Res. Sci. Technol..

[55] Ibekwe A.M., Gonzalez-Rubio A., Suarez D.L. (2018). Impact of treated wastewater for irrigation on soil microbial communities. Sci. Total Environ..

[56] Becerra-Castro C., Lopes A.R., Vaz-Moreira I., Silva E.F., Manaia C.M., Nunes O.C. (2015). Wastewater reuse in irrigation: A microbiological perspective on implications in soil fertility and human and environmental health. Environ. Int..

[57] Abd-Elwahed M.S. (2018). Influence of long-term wastewater irrigation on soil quality and its spatial distribution. Ann. Agric. Sci..

[58] Chen L., Feng Q., Li C., Wei Y., Zhao Y., Feng Y., Zheng H., Li F., Li H. (2017). Impacts of aquaculture wastewater irrigation on soil microbial functional diversity and community structure in arid regions. Sci. Rep..

[59] Wang Y., Wang F., Lu H., Liu Y., Mao C. (2021). Phosphate uptake and transport in plants: An elaborate regulatory system. Plant Cell Physiol..

[60] Chen W., Lu S., Jiao W., Wang M., Chang A.C. (2013). Reclaimed water: A safe irrigation water source?. Environ. Dev..

[61] Al-Absi K.M., Al-Nasir F.M., Mahadeen A.Y. (2009). Mineral content of three olive cultivars irrigated with treated industrial wastewater. Agric. Water Manag..

[62] McDonnell R.P., Staines M.H., Bolland H.S. (2018). Determining the critical plant test potassium concentration for annual and Italian ryegrass on dairy pastures in south-western Australia. Grass Forage Sci..

[63] Bedbabis S., Ferrara G., Ben-Rouina B., Boukhris M. (2010). Effects of irrigation with treated wastewater on olive tree growth, yield and leaf mineral elements at short term. Sci. Hortic..

[64] Batarseh M., Rawajfeh A., Ioannis K., Prodromos K. (2011). Treated municipal wastewater irrigation impact on olive trees (*Olea Europaea* L.) at Al-Tafilah, Jordan. Water Air Soil Pollut..

[65] Alghobar M.A., Suresha S. (2016). Effect of wastewater irrigation on growth and yield of rice crop and uptake and accumulation of nutrient and heavy metals in soil. App. Ecol. Environ. Sci..

[66] United States Salinity Laboratory Staff (1954). Diagnosis and Improvement of Saline and Alkali Soils.

[67] Alloway B.J. (1990). Heavy Metals in Soils.

[68] WHO (2013). Guidelines for the Safe Use of Wastewater and Food Stuff.

[69] Environmental Protection Agency (2007). Water Quality Standards.

[70] Kiziloglu F.M., Turan M., Sahin U., Kuslu Y., Dursun A. (2008). Effects of untreated and treated wastewater irrigation on some chemical properties of cauliflower (*Brassica olerecea* L. var. Botrytis) and red cabbage (*Brassica olerecea* L. var. Rubra) grown on calcareous soil in Turkey. Agric. Water Manag..

[71] Pescod M.B. (1992). Wastewater Treatment and Use in Agriculture.

[72] Eaton A.D., Glescer L.S., Rice E.W., Greenberg A.E. (2005). Standard Methods for the Examination of Water and Wastewater.

[73] Chapman H.D., Parker F. (1961). Determination of NPK, Methods of Analysis for Soil, Plant and Water.

[74] (1990). Official Methods of Analysis.

[75] Steel R.G.D., Torrie J.H., Dickey D.A. (1997). Principles and Procedures of Statistics: A Biometric Approach.

