# Supplementary figures and images for: Nutrients Uptake and Accumulation in Plant Parts of Fragrant Rosa Species Irrigated with Treated and Untreated Wastewater
Source: Plants (Basel). 2022 May 6;11(9):1260. doi: 10.3390/plants11091260 (PMC9103440; doi:10.3390/plants11091260)

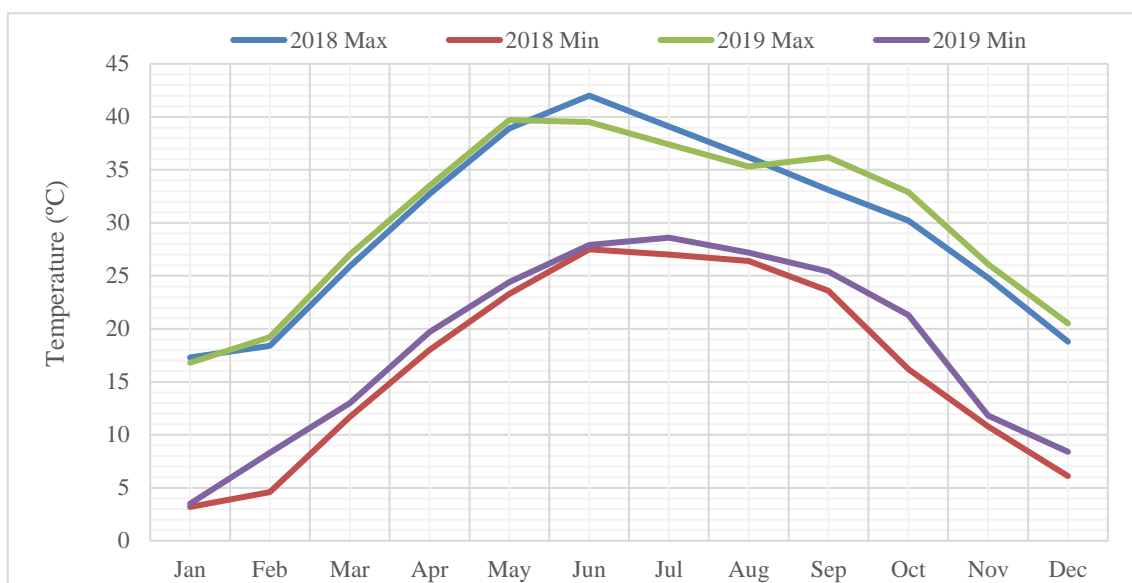

**Figure S1.** Mean monthly minimum and maximum temperature (°C) during the experimental period.

Supplement: Supplementary file 1 [file plants-11-01260-s001.zip › plants-1664653-supplementary.pdf]
